# Multimodal Online Recruitment and Engagement of Black Breast Cancer Survivors for Mixed Methods Social Determinants of Health Research: Feasibility Study

**DOI:** 10.2196/96341

**Published:** 2026-10-07

**Authors:** Kassandra I Alcaraz, Lensa S Keno, Brittany D Jenkins, Avonne E Connor

**Affiliations:** 1 Department of Oncology, School of Medicine, Johns Hopkins University, 401 N Broadway, Baltimore, MD, 21287, United States, 1 410-955-0303; 2 Department of Epidemiology, Bloomberg School of Public Health, Johns Hopkins University, Baltimore, MD, United States; 3 Sidney Kimmel Comprehensive Cancer Center, Johns Hopkins University, Baltimore, MD, United States; 4 Johns Hopkins Center for Health Disparities Solutions, Johns Hopkins University, Baltimore, MD, United States; 5 Department of Biochemistry and Molecular Biology, Bloomberg School of Public Health, Johns Hopkins University, Baltimore, MD, United States

**Keywords:** pilot study, cancer survivors, breast cancer, breast carcinoma, recruitment, engagement, procedures, data collection, biological markers, social determinants of health, health inequities

## Abstract

This pilot study provides evidence that multimodal online recruitment and engagement are feasible for mixed methods social determinants of health research with sociodemographically diverse Black breast cancer survivors.

## Introduction

Black women have the highest breast cancer mortality rate and the shortest overall survival among all racial/ethnic groups in the United States [1], in part due to social determinants of health (SDOH) [2]. However, because racial/ethnic minoritized populations are underrepresented in cancer research, studies examining within-group SDOH and differences among Black breast cancer survivors are limited [3]. Additionally, although some socioeconomically disadvantaged populations have been engaged in research using digital channels [4], evidence indicates that online recruitment methods may not adequately reach diverse sociodemographic groups [5]. Given these limitations, we conducted a preliminary study to assess the feasibility of using online methods to recruit and engage (across several time points) a sociodemographically diverse sample of Black breast cancer survivors. This paper reports on the methods and outcomes of online recruitment and the collection of SDOH-related data using online surveys, virtual focus groups, and biospecimens. Findings provide insights into the processes and feasibility of multimodal online recruitment and engagement for mixed methods SDOH data acquisition.

## Methods

### Overview

From June to September 2025, we recruited adult (age ≥18 years) Black breast cancer survivors residing in Baltimore, Maryland, and 3 surrounding counties. Individuals who were not born female, did not speak English, were diagnosed at stage 0, and were in active cancer treatment or completed active treatment <2 years ago were ineligible. Figure 1 summarizes study procedures. Potentially eligible patients (based on electronic health records) who were seen at any of 4 Johns Hopkins Medicine hospitals or clinics within the past 5 years received a recruitment message in their MyChart (Epic Systems Corp) patient portal. Those who did not have an active MyChart account were emailed the recruitment message. We partnered with community-based organizations to disseminate a study flyer via email and to conduct a webinar. Recruitment source was self-reported in the study’s survey.

**Figure 1.**
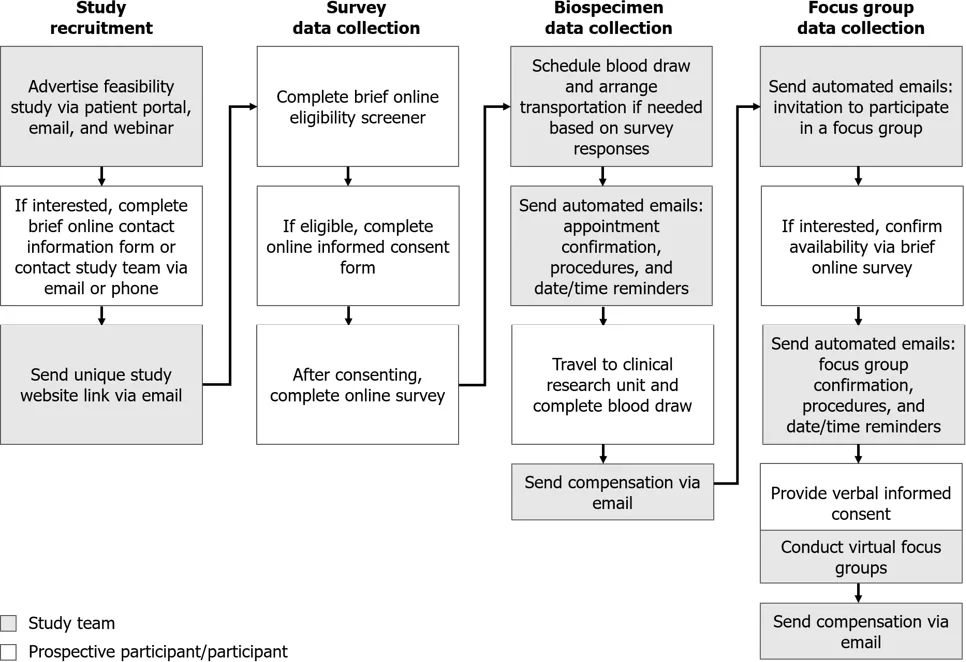
Study processes.

The 20‐ to 30-minute online survey was deployed via REDCap (Vanderbilt University). To enhance internal validity, we aimed to collect biospecimens within 2 weeks of each participant’s survey completion. Respondents were asked to indicate their availability for a blood draw appointment and need for study-arranged transportation assistance via Lyft. Blood draws were completed at the Johns Hopkins ProHealth Clinical Research Unit, where ProHealth staff collected small-volume blood samples (4 mL) and measured participants’ height and weight. Respondents were also asked whether they were interested in participating in one of four 75-minute virtual focus groups via Zoom. Completing the survey was a prerequisite for being scheduled for a blood draw, and completing the blood draw was a prerequisite for being invited to a focus group.

We evaluated feasibility across 3 feasibility framework-guided domains: recruitment, sociodemographic representativeness, and assessment procedures [6,7]. Findings are reported using frequencies and percentages. (Focus group findings are not reported in this paper.) Analyses were performed using SAS (version 9.4; SAS Institute Inc).

### Ethical Considerations

The study received ethics approval from the Institutional Review Board of the Johns Hopkins Bloomberg School of Public Health (IRB00031122). Electronic informed consent was obtained from all participants prior to data collection. Study data were deidentified. Each participant received a US $75 Tango gift card after survey completion and biospecimen donation and a US $50 Tango gift card after focus group participation.

## Results

Our evaluation of recruitment found that 120 (80.0%) out of 150 individuals completed the eligibility screener. Of these 120 participants, 37 (31.0%) were eligible, but 7 (19.0%) of these 37 individuals did not proceed with participation. Among the final sample (n=30), most participants reported learning about the study from the patient portal (Table 1).

**Table 1.** Sample recruitment sources and sociodemographic characteristics (N=30).

Variable	Participants, n (%)
Recruitment source	
Patient portal	25 (83.3)
Email	2 (6.7)
Other (friend/family, online advertisement)	2 (6.7)
Missing	1 (3.3)
Age (years)	
18‐49	5 (16.7)
50‐64	11 (36.7)
≥65	11 (36.7)
Missing	3 (10.0)
Educational attainment	
≤ Some college	9 (30.0)
≥ College graduate	20 (66.7)
Missing	1 (3.3)
Marital status	
Never married	7 (23.3)
Married or partnered	10 (33.3)
Separated, widowed, or divorced	13 (43.3)
Employment status	
Employed	15 (50.0)
Unemployed or retired	14 (46.7)
Missing	1 (3.3)
Annual household income (US $)	
<50,000	9 (30.0)
50,000-99,999	11 (36.7)
≥100,000	8 (26.7)
Missing	2 (6.7)
Number of people in household	
1	12 (40.0)
2	7 (23.3)
≥3	8 (26.7)
Missing	3 (10.0)
Area of residence	
Baltimore City	17 (56.7)
Outside of Baltimore City	13 (43.3)

Our evaluation of sociodemographic representativeness found that a sociodemographically diverse sample was recruited (Table 1), that is, there was a wide variation in participant characteristics. For example, participant age ranged from 43 to 84 years, annual household income ranged from <US $10,000/year to ≥US $200,000/year, and half of the sample were employed.

Our evaluation of assessment procedures found that study assessments were able to be administered as planned. Surveys were completed by all 30 participants, and biospecimens were collected from 27 (90.0%) of the 30. While 29 (97.0%) of 30 participants indicated interest in participating in a focus group, 13 (43.3%) ultimately participated.

## Discussion

### Principal Findings

This study provides evidence on the feasibility of recruiting and engaging a heterogeneous sample of Black breast cancer survivors in SDOH research using online approaches. Online recruitment was feasible, particularly via patient portal. Recruiting a sociodemographically diverse sample and administering assessments also were feasible.

Most participants were recruited from the patient portal, which likely reached a larger number of individuals than other channels. Also, individuals reached through the patient portal may have had greater interest in research participation than email recipients (who were, by definition, nonportal users) or other individuals, potentially due to higher health care system engagement or trust. Overall, study findings align with previous research on the use of digital channels for engaging diverse populations in research. Evidence indicates that digital recruitment approaches, including online recruitment and recruitment using electronic medical records, can enhance representation in health research [8]. Additionally, online strategies to recruit underrepresented groups may benefit from health care provider and/or community partner endorsements [8]. Our future research will seek to enhance community partner–based recruitment.

Despite the availability of transportation assistance, 3 participants did not complete a blood draw; they did not respond to appointment scheduling attempts. Our subsequent research may incorporate approaches such as mobile phlebotomy or self-collection, which may be more convenient and acceptable [9,10]. Additionally, despite high interest, scheduling difficulties (eg, participant work schedules, competing demands) precluded virtual focus group participation among several participants. Our future research may offer additional focus groups or employ one-on-one interviews to facilitate greater scheduling flexibility.

Potential study limitations include the modest sample size, recruitment from a single state, and the sample comprising Black breast cancer survivors only. Therefore, findings may have limited generalizability. Also, the study’s online approach may have disproportionately reached survivors with greater digital access and/or health care system engagement.

### Conclusions

Findings from this feasibility study inform the development of the next phase of our mixed methods research on SDOH and cancer disparities. Results demonstrate the utility of recruiting and engaging sociodemographically diverse research participants using a variety of online methods.

## Abbreviations

SDOH: social determinants of health

## References

[1] Saka AH, Giaquinto AN, McCullough LE (2025). Cancer statistics for African American and Black people, 2025. CA Cancer J Clin.

[2] Alcaraz KI, Wiedt TL, Daniels EC, Yabroff KR, Guerra CE, Wender RC (2020). Understanding and addressing social determinants to advance cancer health equity in the United States: a blueprint for practice, research, and policy. CA Cancer J Clin.

[3] Falcone M, Salhia B, Hughes Halbert C (2024). Impact of structural racism and social determinants of health on disparities in breast cancer mortality. Cancer Res.

[4] Alcaraz KI, Vereen RN, Burnham D (2020). Use of telephone and digital channels to engage socioeconomically disadvantaged adults in health disparities research within a social service setting: cross-sectional study. J Med Internet Res.

[5] Jang M, Vorderstrasse A (2019). Socioeconomic status and racial or ethnic differences in participation: web-based survey. JMIR Res Protoc.

[6] Milbourn H, Campbell A, Clark F (2025). Effectiveness and costs of participant recruitment strategies to a web-based population cohort: observational study. J Med Internet Res.

[7] Aschbrenner KA, Kruse G, Gallo JJ, Plano Clark VL (2022). Applying mixed methods to pilot feasibility studies to inform intervention trials. Pilot Feasibility Stud.

[8] Helm A, Kaufman M, Glassy N (2025). Strategies for recruiting participants underrepresented in clinical research: a scoping review. Soc Sci Med.

[9] McCammon MG, Conrad CM, Klug ZT (2013). From an infrastructure to a service-based business model: 5 years of mobile clinical research at the University of Michigan. Clin Transl Sci.

[10] Schröder D, Müller F, Heesen G (2023). Feasibility of self-organized blood sample collection in adults for study purposes in a primary care setting. PLoS ONE.

